# Bimodal Fucoidan-Coated Zinc Oxide/Iron Oxide-Based Nanoparticles for the Imaging of Atherothrombosis

**DOI:** 10.3390/molecules24050962

**Published:** 2019-03-08

**Authors:** Hoang Nguyen, Eric Tinet, Thierry Chauveau, Frédéric Geinguenaud, Yoann Lalatonne, Aude Michel, Rachida Aid-Launais, Clément Journé, Caroline Lefèbvre, Teresa Simon-Yarza, Laurence Motte, Noureddine Jouini, Jean-Michel Tualle, Frédéric Chaubet

**Affiliations:** 1 Laboratory for Vascular Translational Science, Inserm U1148, Institut Galilée—Université Paris Diderot, Université Paris 13, Sorbonne-Paris-Cité, 99 av JB Clément, 93430 Villetaneuse, France; ndhoang@iop.vast.ac.vn (H.N.); frederic.geinguenaud@univ-paris13.fr (F.G.); yoann.lalatonne@aphp.fr (Y.L.); rachida.aid@inserm.fr (R.A.-L.); clement.journe@inserm.fr (C.J.); teresasimonyarza@gmail.com (T.S.-Y.); laurence.motte@univ-paris13.fr (L.M.); 2 Laboratoire de Physique des Lasers, UMR CNRS 7538, Institut Galilée—Université Paris 13, Sorbonne-Paris-Cité, 99 av JB Clément, 93430 Villetaneuse, France; eric.tinet@univ-paris13.fr (E.T.); tualle@univ-paris13.fr (J.-M.T.); 3 Laboratoire des Sciences des Procédés et des Matériaux, UPR CNRS 3407, Institut Galilée—Université Paris 13, Sorbonne-Paris-Cité, 99 av JB Clément, 93430 Villetaneuse, France; thierry.chauveau@univ-paris13.fr (T.C.); jouini@univ-paris13.fr (N.J.); 4 Service de Médecine Nucléaire, Hôpital Avicenne Assistance Publique-Hôpitaux de Paris, F-93009 Bobigny, France; 5 Laboratoire Phénix, UMR 8234, UPMC, 4 place Jussieu, 75252 Paris Cedex 05, France; aude.michel@upmc.fr; 6 Fédération de Recherche en Imagerie multimodalité (FRIM), UMS 34, Hôpital Bichat, 46 rue Henri Huchard, 75018 Paris Cedex, France; 7 Université de Technologie de Compiègne, Service d’Analyse Physico-Chimique, Direction à la Recherche, Rue du Dr Schweitzer, CS 60319, 60203 Compiègne cedex, France; caroline.lefebvre@utc.fr

**Keywords:** zinc oxide, iron oxide, nanoparticles, fucoidan, atherothrombosis, MRI, optical imaging, contrast agents

## Abstract

A polyol method was used to obtain ultrasmall ZnO nanoparticles (NPs) doped with iron ions and coated with a low molecular weight fucoidan in order to perform in vivo MR and ex vivo fluorescence imaging of athrothrombosis. During the synthesis, the early elimination of water by azeotropic distillation with toluene allowed us to produce NPs which size, determined by XRD and TEM, decreased from 7 nm to 4 nm with the increase of iron/zinc ratios from 0.05 to 0.50 respectively. For the highest iron content (NP-0.50) NPs were evidenced as a mixture of nanocrystals made of wurtzite and cubic phase with a molar ratio of 2.57:1, although it was not possible to distinguish one from the other by TEM. NP-0.50 were superparamagnetic and exhibited a large emission spectrum at 470 nm when excited at 370 nm. After surface functionalization of NP-0.50 with fucoidan (fuco-0.50), the hydrodynamic size in the physiological medium was 162.0 ± 0.4 nm, with a corresponding negative zeta potential of −48.7 ± 0.4 mV, respectively. The coating was evidenced by FT-IR spectra and thermogravimetric analysis. Aqueous suspensions of fuco-0.50 revealed high transverse proton relaxivities (T_2_) with an r_2_ value of 173.5 mM^−1^ s^−1^ (300 K, 7.0 T) and remained stable for more than 3 months in water or in phosphate buffer saline without evolution of the hydrodynamic size and size distribution. No cytotoxic effect was observed on human endothelial cells up to 48 h with these NPs at a dose of 0.1 mg/mL. After injection into a rat model of atherothrombosis, MR imaging allowed the localization of diseased areas and the subsequent fluorescence imaging of thrombus on tissue slices.

## 1. Introduction

Cardiovascular diseases (CVD) are the leading cause of death in the world [1]. The main expression of CVD is atherosclerosis which consists of the formation of a plaque mainly at the inner surface of the arterial vascular wall [2]. Massive plaque rupture or arterial intraplaque haemorrhage induce a thrombus formation (namely atherothrombosis) leading to dramatic consequences such as acute coronary syndrome and stroke. Medical imaging is the most widespread tool for the diagnosis of atherothrombosis, molecular imaging being a promising development dedicated to the visualization of the biological processes at the cellular and molecular levels [3]. Among the various molecular imaging modalities, MRI is by far the most used and considered a gold standard in the clinic for detecting atherothrombosis. However, the spatial resolution of a commercial MRI machine is about one hundred micrometers [4,5,6] and this resolution requires a long acquisition time. This limited resolution is not suitable for analyzing the distribution of the MRI agents in small volumes such as a single cell or the layers of a small artery wall. Biospecific contrast agents allowing both MR and optical imaging would be relevant tools for preclinical studies, since optical microscopy, which offers a much higher spatial resolution than MRI (~100×), can be used on MRI-localized diseased tissue sections [7]. Many efforts to synthesize fluorescent contrast agents were proposed in order to advance the art, as for example, fluorophores linked with MRI agents [6,8]. Semiconductor quantum dots are the most investigated luminescent nanomaterials, owing to their advantages over the widespread organic dyes. Among them, ZnO is widely used as an industrial additive to produce a lot of materials mainly thanks to unique magneto-optic properties [9,10,11]. ZnO is widely admitted as a non-toxic material since there is no evidence of carcinogenicity, genotoxicity and reproduction toxicity in humans [12]. In addition to luminescence properties related to quantum size effect, the Fe-doping ZnO NPs (Zn(Fe)O) would allow for the consideration of dual MR and optical bimodal imaging. Zn(Fe)O have been synthesized by many ways [13], the most popular approach being the polyol method [14] which makes it possible to solubilize a large number of metallic precursors and to efficiently activate the reactions favoring particles with a high crystallinity and a narrow size distribution [15,16] and Fe_3_O_4_@ZnO [17,18], and ZnO nanorods decorated with γ-Fe_2_O_3_ have been proposed [19]. The formation of Zn(Fe)O nanocrystals without modification of the elementary cell, i.e., leading to the same crystal lattice, could be obtained either by substitution of some zinc ions by iron ions, or by the inclusion of iron ions into tetrahedral sites of the hexagonal wurtzite cell [20,21]. ZnO-based nanocrystals doped with up to 25 wt% Fe synthesized by the polyol method have been reported with a size of 17 nm and a narrow size distribution [20]. Eventually, a drastic reduction of the toxicity of iron-doped ZnO by comparison with pure ZnO nanocrystals was observed in rodent and zebrafish models up to an iron content of 10 wt% Fe [22].

In a thrombotic situation, activated platelets and inflamed endothelium overexpress specific membrane glycoproteins, in particular, P-selectin, which is recognized by its main ligand, P-selectin glycoprotein ligand-1 (PSGL-1), expressed by lymphocytes [23]. Fucoidan, heparin and dextran sulfate are sulfated polysaccharides which bind to P-selectin [24] mimicking the interaction with PSGL-1. Low molecular weight fucoidan was found as the most efficient glycosidic ligand of P-selectin in a purified system and in human whole blood experiments [24,25,26,27,28,29]. In this context, P-selectin appears as a good candidate target for the diagnosis of atherothrombosis and a contrast agent able to bind to P-selectin, i.e., vectorized by fucoidan to activated platelets, would efficiently enhance the detection of early thrombus formation.

In the present study, we propose a novel approach for molecular imaging of thrombus using fucoidan coated on ultrasmall superparamagnetic and luminescent Zn(Fe)O hybrid nanoparticles. We have evaluated the ability of this new contrast agent to detect atherothrombosis in a rat model, thanks to the magnetic properties of the NPs, before optical examination with a dedicated microscope of thin slices of diseased tissues by fluorescence under ultra-violet (UV) excitation.

## 2. Results and Discussion

### 2.1. Composition and Structure of Nanoparticles

NPs have been obtained from zinc and iron acetates in diethylene glycol (DEG) after elimination of water from the medium with toluene (experimental setup is provided in Figure S1). The composition, size and lattice parameters are gathered in Table 1. NPs were defined by their starting iron content R_Fe,I_ = Fe/(Fe + Zn) = 0.05, 0.15, 0.35 and 0.50 (labeled NP-0.05, NP-0.15, NP-0.35 and NP-0.50 respectively). The final Fe contents (R_Fe,f_), determined by atomic absorption spectroscopy were higher but quite close to those of the starting ones. The size of NPs and the phase composition and crystal structure were obtained from electronic microscopy (TEM), energy-dispersive X-ray spectroscopy (EDS), elemental analysis and X-ray diffraction (XRD) analysis.

**Figure 1:**
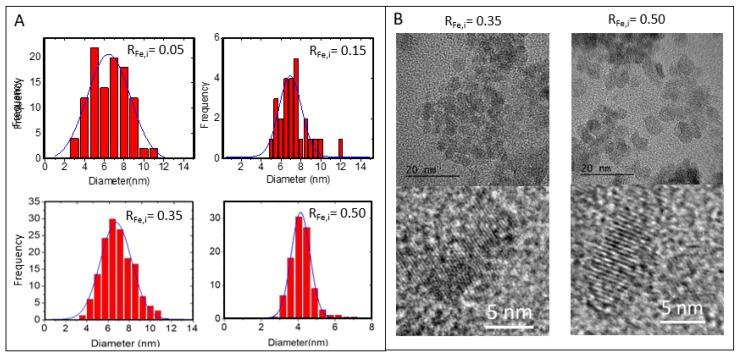
(**A**) Size distributions of NPs obtained from Image J processing of the Transmission Electronique Microscopy (TEM) images from 200 NPs, (**B**) High-Resolution TEM (HR-TEM) images of NP-0.35 (R_Fe,i_ = 0.35) and NP-0.50 (R_Fe,i_ = 0.50) with corresponding enlargements evidencing the crystalline planes.

**Figure 2:**
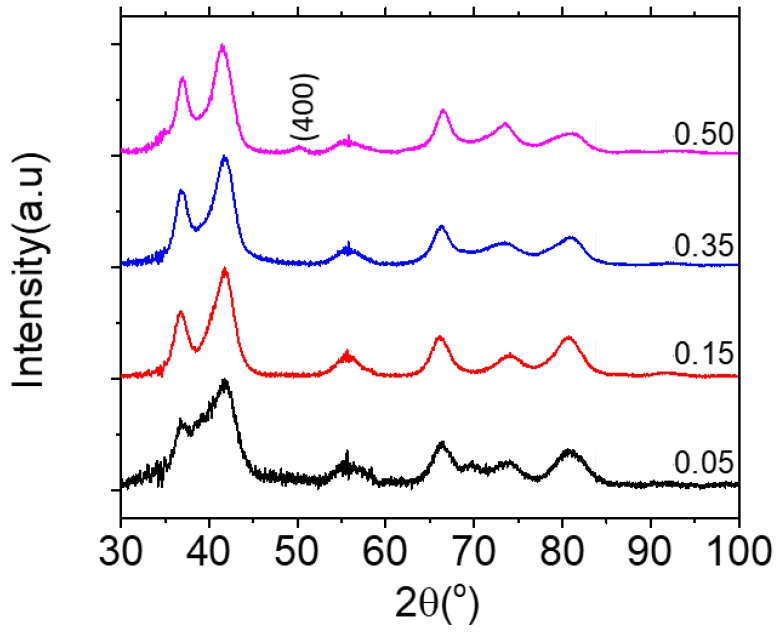
XRD of Zn(Fe)O NPs-0.05, NP-0.15, NP-0.35 and NP-0.50 (from bottom to top). The signal appearing in the 46°–53° area on the top diffractogram corresponds to a cubic phase indexation (400).

TEM and size distribution are presented in Figure 1. It can be observed that by increasing R_Fe,i_, NPs diameters decreased from 7.0 ± 0.8 nm to 4.2 ± 0.6 nm. High-resolution TEM (HR-TEM) images of NP-0.35 and NP-0.50 shows highly crystalized single crystal and EDS analysis evidenced qualitatively the presence of iron and zinc ions (Figure S2). XRD patterns in Figure 2 consist of wide peaks, which confirms the nanocrystalline nature of the prepared material. The crystal size obtained from Rietveld refinement analysis performed using MAUD (material analysis using diffraction) software [30] is in accordance with the values from TEM as well as the stoichiometry established from chemical analysis. For NP-0.05, NP-0.15 and NP-0.35 a wurtzite structure with an increase of iron content is evidenced. Concerning NP-0.50, the formation of an additional ZnFe_2_O_4_ spinel phase is observed (Table 1).

The formation of Zn(Fe)O crystals without modification of the elementary cell, i.e., leading to the same crystal lattice, is obtained either by substitution of some zinc ions by iron ions or by inclusion of iron ions into tetraedric sites of the hexagonal cell [20,21]. For NP-0.50, we tried to propose a stoichiometry for the hexagonal phase by making the hypothesis of substitution. On diffractograms, only the peaks in the angular domains 17–25°, 46–53° and 59–65° could be unequivocally assigned to the ZnFe_2_O_4_ compound. However, due to the very small size of the coherence domains, the peaks 17–25° and 59–65° result from the convolution of the peaks of the two phases whose deconvolution is difficult. Compared with the other preparations, the additional signal between 46° and 53°, which corresponds to an indexation (400) of the cubic phase, made it possible to conclude that the two Zn_0.54_(Fe)_0.46_O and ZnFe_2_O_4_ phases were mixed with a molar proportion determined by refinement of 2.57: 1. In previous works using polyol method the NPs sizes were higher than those obtained in our study: Balti et al.: 17 nm [20], Dinesha et al.: 19–34 nm [21], Ciciliati et al.: 11–25 nm [31], Il’ves et al.: 10–20 nm [13] and Arciniegas-Grijalba et al.: 20–35 nm [32]. Moreover, irrespective of the synthesis method, other studies have observed that the size of Zn(Fe)O NPs decreased with the increase of iron concentration and they noted also the formation of a secondary phase-spinel ZnFe_2_O_4_ increasing with the concentration of Fe doping and with the annealing temperature [13]. Surprisingly, TEM evidenced a unique population of NP-0.50 with an average size of 4.2 ± 0.6 nm in accordance with the sizes of the crystallites determined by XRD (Table 1). The rate of particle growth is governed by hydrolysis and condensation reactions and by the concentration of the precursors and their reactivity, which depends on the number of particle surface atoms, and the solution composition [33,34]. Poul et al. reported that the ZnO particle sizes tend to become smaller with decreasing the water content of the reaction medium [35]. The elimination of water by the use of toluene occurred over one hour at a temperature of 110 °C. We hypothesize that for this time, nucleation occurred but the growth of wurtzite crystals was very limited by the rapid removal of water molecules. The formation of other secondary phases, such as Fe_3_O_4_, could also be observed which was not the case in our study, likely due to the relatively low temperature of the polyol medium as compared to much higher annealing temperatures needed to induce the formation of Fe_3_O_4_ [13].

### 2.2. NP Coating with Polysaccharides

**Figure 3:**
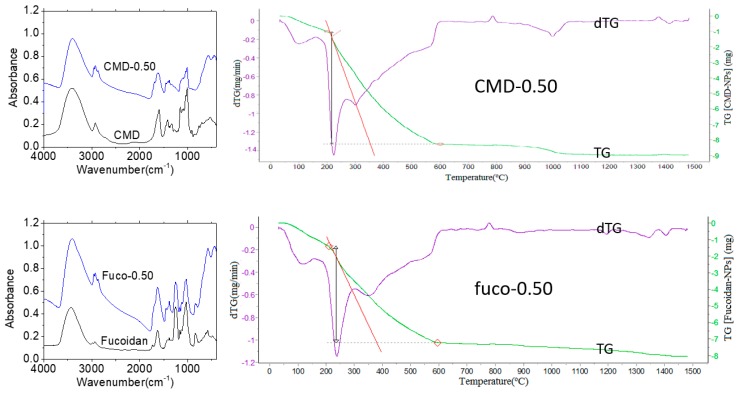
FT-IR absorption spectra and thermograms of fucoidan coated NP-0.50 (fuco-0.50) and carboxymethyldextran (CMD) coated NP-0.50 (CMD-0.50). On thermograms, red diamonds point out water loss and the total degradation of the NPs’ organic layer (see text).

NPs-0.50 were considered as a single population and coated with two negatively charged polysaccharides, carboxymethyldextran (CMD-0.50) as control and fucoidan (fuco-0.50) as P-selectin targeting moiety, using a precipitation-redispersion mechanism for the direct and one-step complexation and taking care of avidity effect of carboxylate and sulfate groups for oxide surface [36]. After surface functionalization, the hydrodynamic sizes in the physiological medium were 177.0 ± 0.4 nm and 162.0 ± 0.4 nm, with corresponding negative zeta potential of −33.3 ± 0.4 mV and −48.7 ± 0.4 mV, respectively. The coatings were evidenced by FT-IR spectra and thermogravimetric analysis (TGA) (Figure 3).

**Figure 4:**
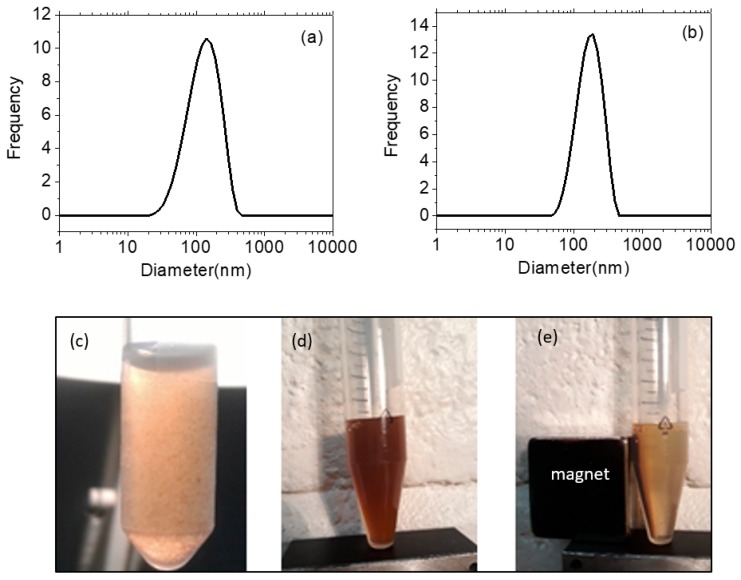
Size and stability of the colloids. Size distribution of fuco-0.50 (**a**) and CMD-0.50 (**b**) in water. Suspension of bare NP-0.50 at the concentration of 5 mg/mL (**c**), fuco-0.50 (**d**), and fuco-0.50 with applied magnetic field (**e**) in water ([fuco-0.50] = 20 mg/mL).

Comparison of FT-IR spectra of coated NPs with those of the native polysaccharides evidenced the organic layer around the NPs. Both thermograms exhibit a two-step decomposition: (i) the removal of water up to 200 °C (CMD-0.50) and 208 °C (fuco-0.50) estimated to about 10% (*w*/*w*) and (ii) the consecutive full degradation of the organic layer ending at 601 °C (CMD-0.50) and 595 °C (fuco-0.50). The overall weight losses (37.4% and 48.8% (*w*/*w*) for CMD-0.50 and fuco-0.50 respectively) allowed to estimate an average quantity of molecules per nanoparticle of about 30 for CMD and for fucoidan, by considering an average density of 5.0 g/cm^3^ which is commonly admitted for ZnO-based NPs. This was in accordance with the average amount of fucoidan estimated about 30 molecules/NP from the sulfate analysis of fuco-0.50 by comparison with the sulfate composition of the starting fucoidan [28]. To our knowledge, this was the first direct coating of NPs with fucoidan since an intermediate carboxymethyldextran layer was used in previous works [26,28]. The bare NPs were stable in ethanol, but a quick aggregation and precipitation were observed in a few hours in water. Conversely, the suspension of fuco-0.50 remained stable for more than 3 months in water or in phosphate buffer saline (Figure 4) without evolution of the hydrodynamic size and size distribution.

### 2.3. Magnetic Properties

**Figure 5:**
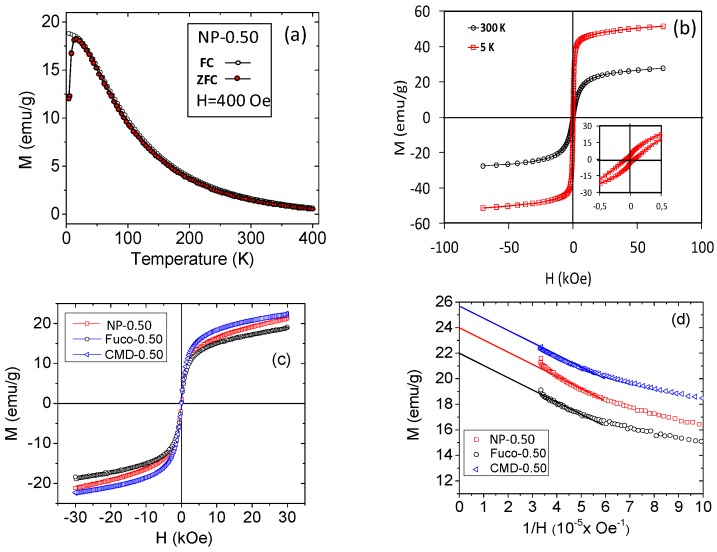
Magnetic properties of bare and coated NP-0.50. Upper row: Magnetization (M) versus temperature (**a**) and M vs. applied field (H) at 5K and 300 K (**b**) of NP-0.50. Lower row: M vs. applied field (H) in water at 300 K (**c**), and M vs. 1/H plot for the determination of M_S_ for NP-0.50; CMD-0.50 and Fuco-O.50 (**d**).

The zero-field-cooled (ZFC) and the field-cooled (FC) magnetization curves measured in the low applied magnetic field of 400 Oe are shown in Figure 5a. The ZFC curve gives information about the ferri-superparamagnetic transition of the system, which occurs at the temperature of the maximum magnetization value, the blocking temperature (T_B_). For our system, a T_B_ value of 10 K was obtained. Furthermore, the magnetic properties of NP-0.50 were determined at 5 K and 300 K when the applied field ranged from −70 to +70 kOe (Figure 5b). At 5 K, the NP-0.50 show ferrimagnetic behavior with a coercivity of about 70 Oe and a magnetization saturation Ms equal to 48 emu/g at 70 kOe. At 300 K, the NP-0.50 become superparamagnetic because of size effects and thermal fluctuations [37,38]. Moreover, the magnetization saturation is reduced to about 24 emu/g (Figure 5d). After coating with fucoidan or CMD, superparamagnetic behavior is maintained (Figure 5c) with no change in magnetization saturation (Figure 5d).

**Figure 6:**
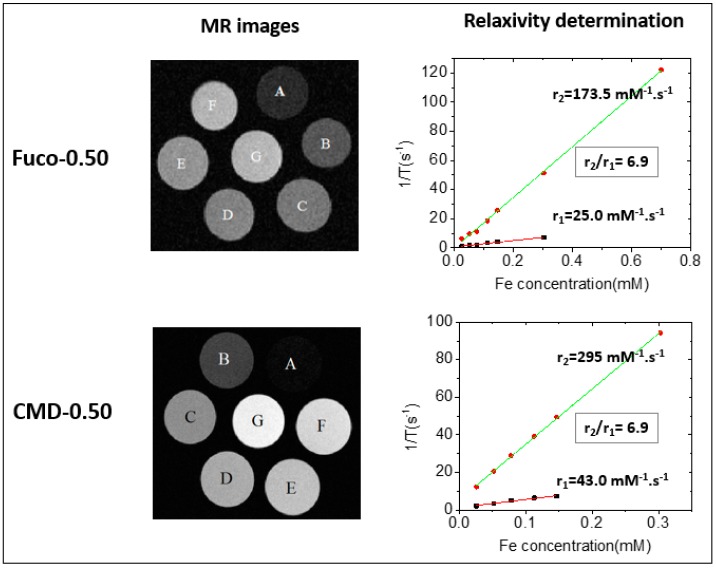
MRI phantoms and relaxivity determination from 1/T vs. Fe concentration curves for fuco-0.50 and CMD-0.50 samples, with C_Fe_ = 0.7(A), 0.303(B), 0.147(C), 0.113(D), 0.0774(E), 0.0522(F), and 0.0261(G) mM. For CMD-0.50, the plotted data are ranging from C_Fe_ = 0.303 mM (B) to 0.0261mM (G).

Longitudinal r_1_ and transverse r_2_ relaxivities of fuco-0.50 and CMD-0.50 in water were measured by dedicated T_1_ and T_2_ mapping sequences using a 7 T MRI preclinical scanner. The relaxation rates of NPs in suspension were obtained by linear fitting relaxation rates (1/T_1_) and (1/T_2_) versus different Fe concentrations. For CMD-0.50 and fuco-0.50, r_2_ relaxivities were 295 mM^−1^ s^−1^ and 173.5 mM^−1^ s^−1^ whereas r_1_ relaxivities were 43.0 mM^−1^ s^−1^ and 25.0 mM^−1^ s^−1^ respectively (Figure 6).

As it can be observed, hyposignal increased when increasing NPs concentration in phantoms, demonstrating in vitro the MRI contrast properties of fuco-0.50 and CMD-0.50. The decrease in transverse relaxivity observed with the fucoidan-coated NPs compared to the CMD coated NPs can be related to the decrease in hydrodynamic size, 162 nm and 177 nm respectively, leading to a slower Brownian NP rotation [39]. In parallel, it is observed a decrease of longitudinal relaxivity values. Considering that T1 performance is related to the exposure of metal ions at the NP surface and the water accessibility, this decrease of the r1 value observed with the fucoidan-coated NPs compared to the CMD coated NPs, has to be related to lower complexation of iron ions with sulfate groups of fucoidan compared to carboxylate groups of CMD. This concomitant decrease in longitudinal and transverse relaxivities leads to a constant r_2_/r_1_ ratio equal to 6.9 for CMD-0.50 and fuco-0.5. CMD-coated and fucoidan-coated NPs exhibited rather high values compared to other studies with iron oxide NPs [40,41,42,43,44,45] and ZnFe_2_O_4_ NPs [46,47,48] and to the reported relaxivities of commercially available T_2_ contrast agents such as Resovist (r_2_ = 177 mM^−1^ s^−1^ at 7 T) and Endorem (r_2_ = 160 mM^−1^ s^−1^ at 7 T) [39].

### 2.4. Optical Properties

**Figure 7:**
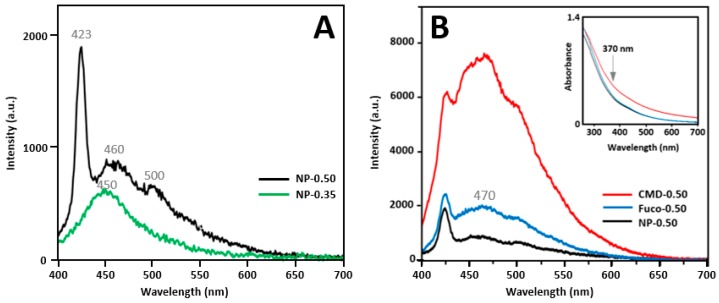
(**A**) Fluorescence spectra of NP-0.50 and NP-0.35 with λ_exc_ = 370nm, at 2mg/mL in ethanol; (**B**) Fluorescence spectra of NP-0.50 (in ethanol) and capped NPs (in water) at 2.0 mg/mL, absorption spectra of the samples are provided in the insert. Grey numbers correspond to wavelength maxima (see text).

There are mainly three factors to be considered when ZnO-based NPs are used as fluorescent markers in biological media: (i) the protect coating should be biocompatible to the tissues and cells; (ii) the luminescence emission of NPs should not be covered by the autofluorescence of the biological background; and (iii) the luminescence should not be quenched by surface modification [11]. Figure 7 presents the emission spectra of NP-0.35 and NP-0.50 (7A), and fluorescence and absorption spectra of bare and coated NP-0.50 (7B). The excitation wavelength was 370 nm. Note that neither fucoidan nor CMD presented a significant absorption in the visible domain (Figure S3). The luminescence of ZnO-based nanocrystals is currently observed with an excitation wavelength in the 250–370 nm region depending on the preparation method and the surface properties [49,50]. Although there was no evident absorption peak in this region, 370 nm seemed a good compromise between the intensity of the emission at 470 nm and the need to avoid further alteration of the tissue slices from high energy beam (see § 2.6). The NP-0.50 sample had a higher overall luminescence than NP-0.35. A wide emission band centered on 450 nm was observed for NP-0.35 (wurtzite Zn(Fe)O crystals). For NP-0.50, this band was more intense and shifted to about 460 nm with a shoulder at 500 nm and a new sharp emission band at 423 nm appeared. These signals could be attributed to a ZnFe_2_O_4_ phase [50] which constituted 28% (mol/mol) of the nanocrystals of the NP-0.50 sample as evidenced by XRD analysis. Wavelengths shifts and variations of the intensities of the emission bands are due (i) to various intrinsic defects in oxides such as oxygen vacancies, singly ionized oxygen vacancies, antisite oxygen, zinc vacancies and even oxygen surface defects [51,52,53] and (ii) to the passivation of some defects by the capping polymers [49]. Adequate coating allows the particles to stabilize sterically in the biological medium [11], but depending on the polymers used, the luminescence properties can be reduced [49] or improved [54]. CMD and fucoidan are clearly in the second category, favoring their use in bioimaging as evidenced in Figure 7B. The large difference in emission intensity between these two samples in favor of the CMD-0.50 could be explained by a greater affinity of the carboxylate functions with the surface of the nanoparticles with respect to the sulfate groups of fucoidan. The formation of ZnFe_2_O_4_ was previously reported during the preparation of iron-doped ZnO NPs as well as a luminescence quenching of doped NPs by ferrite nanocrystals [50,55,56]. Indeed, although emission spectra evidenced a large visible signal with an excitation wavelength of 370 nm, the intensity of the luminescence remained weak in spite of some exaltation by the coatings. Srivastava et al. attributed part of this quenching to a boundary energy transfer between co-prepared species [50,57]. However, in our study NPs were obtained as well separated nanocrystals. Because of their identical size, the coating with fucoidan and CMD likely occurred in the same way for both types of nanocrystals and the final suspension would consist of a mixture, ferrite being mainly responsible for luminescence. Eventually, NP-0.50 samples appeared as the best compromise, since magnetic properties allowing in vivo MR imaging localization of the diseased tissue thanks to fucoidan targeting had to be first taken into account.

### 2.5. Cytotoxicity

**Figure 8:**
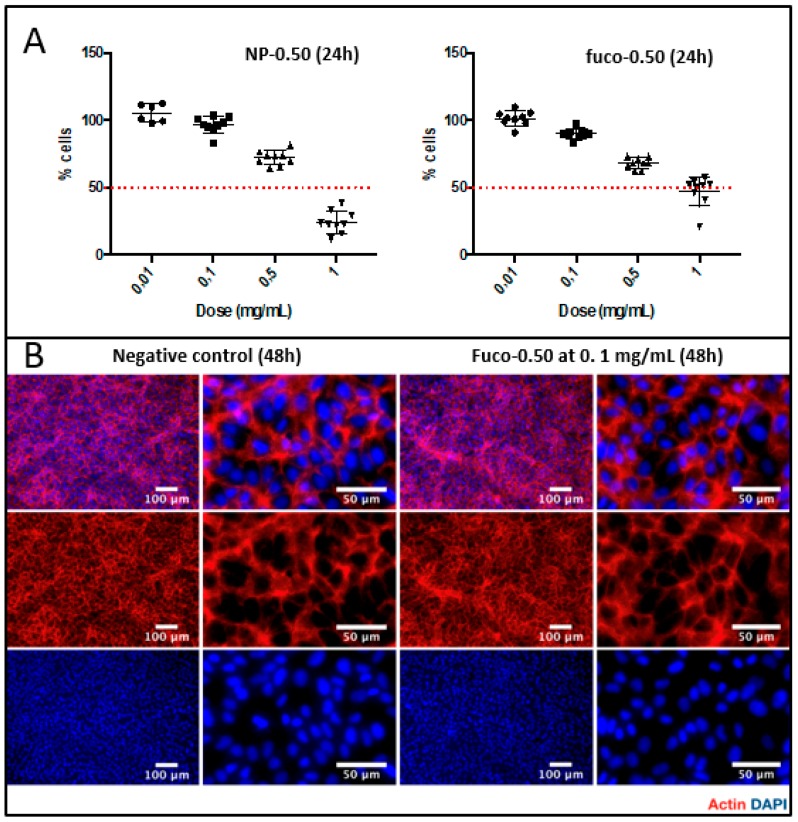
(**A**) MTT proliferation assay of NP-0.50 and fuco-0.50 (mg/mL) toward human vascular endothelial cells (HUVECs) after 24 h. (**B**) Fucoidan-coated NPs effect on cell morphology and HUVECs cytoskeleton organization after 48 h at a dose of 0.1 mg/mL.

The proliferation and the morphology of human endothelial cells, as well as the cytoskeleton organization, were assayed in contact with NP-0.50 and fuco-0.50 (Figure 8). Up to a concentration of 0.1 mg/mL, neither product limited cell proliferation over 24 h. Fuco-0.50 at a dose of 0.1 mg/mL did not alter cell morphology after 48 h as compared to the untreated cells. Cytotoxicity of the samples was similar over 72 h up to 0.1 mg/mL. At 24 h and 48 h, fucoidan-coated NPs were observed less toxic than the bare ones whatever the concentration. At 72 h, more than 50% cells died for concentrations above 0.1 mg/mL (Figure S4).

### 2.6. MR and Optical Imaging

**Figure 9:**
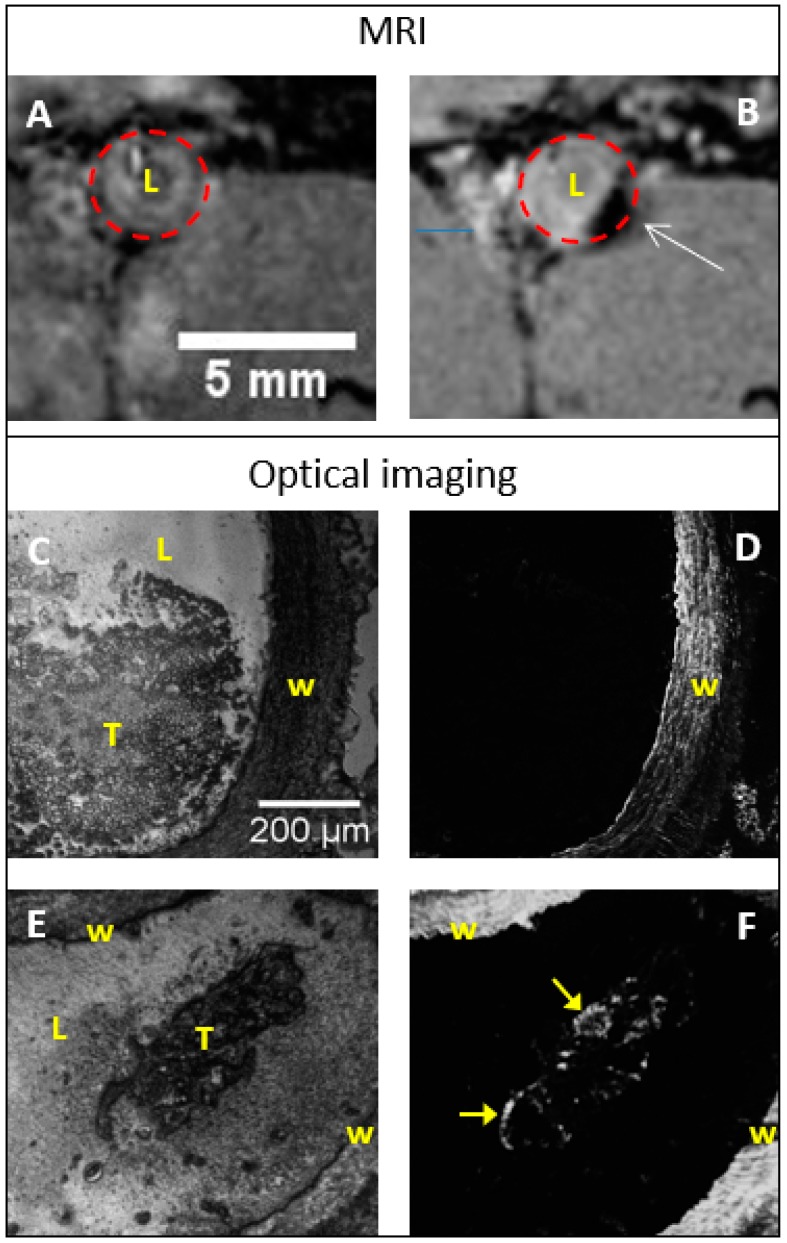
MR and optical images of rat abdominal aorta aneurysm (AAA). Top row: transversal MR images before (**A**) and 2 h after (**B**) injection of fuco-0.50. The white arrow points out the thrombosed area. Time course of the contrast over 2 h is provided in Figure S6. Middle row: histological slices from AAA without injection of fuco-0.50; (**C**) = bright field imaging; (**D**) = fluorescence imaging. Bottom row: histological slices from AAA after injection of fuco-0.50; (**E**) bright field imaging; (**F**) fluorescence imaging. The yellow arrows point out fluorescent areas on thrombus. Caption: W = vascular wall; L = Lumen; T = thrombus. Fluorescence images were recorded at 450 nm with λ_ex_ = 370 nm.

Fuco-0.50 and CMD-0.50 have been intrevenously injected in a rat model of aneurysmal thrombosis with a dose of 200 µmoles Fe per kg (i.e., about 5.3 mM Fe, see Experimental) as previously described [26]. MRI allowed the localization of the diseased areas which were harvested after the sacrifice of the animals and histological sections were prepared for further optical imaging with a dedicated fluorescence microscopy setup (Figure S5). The difference of MR images before and after injection with coated NPs evidenced the uptake of fuco-0.50 into the diseased tissue appearing as a contrasted black area (Figure 9).

Fucoidan coated NPs were linked to the thrombus likely through an interaction with P-selectin overexpressed by aggregated platelets [24] as evidenced by Suzuki et al. with fucoidan-coated USPIO [26]. Two hours after injection, the concentration of NPs in the thrombus reached the maximum level, corresponding to a minimum of the MR signal (Figure S6). Moreover, MRI kinetics was performed on elastase-treated animals with sequential injections of CMD-0.50 and fuco-0.50 into the same animal (Figure S7). No signal change was observed after CMD-0.50 injection for 3 h, but a significant hyposignal was highly visible on the same animal between 30 and 60 min after injection of fuco-0.50 (with a small thrombus confirmed by histology). A strong hyposignal in the aorta was observed only after fuco-0.50 injection. After injection of CMD-0.50 or fuco-0.50, thrombosed aortas were harvested, fixed and cut to get histological slices which were examined by optical imaging.

Luminescence at 470 nm with an excitation wavelength of 370 nm could be recorded with 2.0 mg/mL of NPs suspensions, corresponding to 6.9 µmoles Fe/mL (6.9 mM Fe). If we consider that mainly ZnFe_2_O_4_ contributed to the luminescence of the suspension this estimation must take into account the molar ratio of the two different species (i.e., 1 ZnFe_2_O_4_ for 2.57 Zn_0.46_Fe_0.54_O) and the iron concentration needed for an efficient luminescence should be 6.9/0.28 = 25 µM, i.e., almost 5 times the injected concentration (5.3 mM Fe). However, we must keep in mind that, in one hand, once injected, the fucoidan-coated NPs tend to accumulate into the diseased area as demonstrated by MRI, and in another hand, we don’t know the actual amounts of NPs reaching the area. The luminescence of NPs was weaker than that of tissues and the distribution of NPs was rather difficult to obtain even with a dedicated optical system. Autofluorescence of collagen evidenced the vessel wall in both cases (Figure 9C–F) while some new bright areas on the thrombus could be attributed to large concentrations of fucoidan-coated NPs (Figure 9E,F).

## 3. Experimental

### 3.1. Chemicals

Zinc acetate dehydrate (98%, Sigma Aldrich, Lesquin, France) and iron II acetate (97%, Strem Chemicals, Newburyport, MA, USA) were used as precursors. Diethylene glycol (99%, Alfa Aesar, Haverhill, MA, USA), and toluene (99%, Alfa Aesar) were used as a solvent. Carboxymethyl dextran was purchased from Sigma Aldrich (MW = 10–20 KDa, and CM content = 1.1–1.5 mmol CM/g). Low molecular weight fucoidan from the brown seaweed Ascophyllum nodosum was purchased from Algues & Mer (Ile d’Ouessant, France). Fucose and sulfate contents were 43.0 ± 0.2 and 26.5 ± 0.2 respectively (%*w*/*w*) with Mn = 7200 g/mol and Mw = 10,100 g/mol. Purified water used for the experiments was obtained by ion-exchange from running tap water (Millipore, Guyancourt, France).

### 3.2. Preparation of NPs

It should be first noted that, as much as possible, the NPs have been maintained suspended in a liquid phase. Very small samples were dried only to estimate yields (always above 70%) and to perform some analyzes (such as XRD and thermogravimetry (TGA)) requiring dried powders. The concentration of the suspensions was determined by atomic absorption spectroscopy (see below). There were two reasons for this strategy: NPs in the dry state are very difficult to resuspend in liquids and their potential toxicity to the manipulator is limited in the liquid phase.

#### 3.2.1. Bare NPs

0.02 mol of zinc acetate and iron II acetate with different Fe/Zn ratio (mol/mol) (R_Fe,i_ = Fe/(Zn + Fe) = 0, 0.05, 0.15, 0.35 and 0.50) were dried at 60 °C under vacuum overnight. Then salts were dispersed in a mixture of 80 mL of diethylene glycol (DEG) and 120 mL of toluene, in a three neck 500 mL round bottom flask and heated under nitrogen and a gentle mechanical stirring (Figure S1). The flask was connected to a Dean-Stark apparatus for entrapping water azeotropically. Water (a few mL) and toluene were successively collected by reflux into the Dean-Stark trap and fully eliminated through a Teflon tap. During this process, the temperature did not exceed 110 °C. All toluene was collected in 1 h and the temperature started to rise again reaching DEG reflux (245 °C) in a few minutes. The mixture was maintained at 245 °C for four additional hours. After cooling down to room temperature, NPs were quantitatively collected by centrifugation (8000 rpm, 15 min) and washed two times with acetone, 2 times with ethanol and kept as a suspension in absolute ethanol.

#### 3.2.2. Fucoidan Coating

A 3 mL suspension was prepared as following: 1 mL of DEG-NPs (R_Fe_ = 0.05, 0.15, 0.35, 0.50) in HCl 10-2M ([Fe] = 0.12 mol/L) was diluted in 14 mL of HCl 10-2 M (pH = 2) and sonicated for 30 min (A). Then 4 mg of fucoidan was dissolved in 15 mL of water (B). A and B were mixed and gently shaken at room temperature for 2 h. The fucoidan coated NPs were collected by centrifugation (7300 rpm, 30 min) and resuspended in 3 mL solution of glucose 5% (*w*/*w*). The pH of the final solution was adjusted to 7.2 and 7.5 with a few drops of diluted NaOH. The suspension was split into six tubes of 0.5 mL each which were kept in the dark at −20 °C.

#### 3.2.3. Carboxymethyl Dextran Coating

Six hundred and sixty seven microliter of NPs in HCl 10-2M ([Fe] = 0.12 mol/L) was diluted in 10 mL of HCl 10-2 M (pH = 2) and sonicated for 30 min (C). Then 3.5 mg carboxymethyl dextran sodium salt (CMD) was dissolved in 10 mL of water (D). C and D were mixed and gently shaken at room temperature for 2 h. The CMD-coated NPs were collected by centrifugation (7300 rpm, 30 min) and resuspended in 2 mL solution of glucose 5% (*w*/*w*). The pH of the final solution was adjusted to 7.2 and 7.5 with a few drops of diluted NaOH. The suspension was split into 4 tubes of 0.5 mL each which were kept in the dark at −20 °C.

### 3.3. Iron and Zinc Concentration

The iron and zinc compositions were obtained from suspension (0.5 M) by atomic absorption spectroscopy (AAS) with a Perkin-Elmer Analyst 100 apparatus (Perkin-Elmer, Villebon-sur-Yvette, France) after degradation of the nanocrystals in boiling HCl (35%).

### 3.4. Fucoidan Content

The fucoidan coating was evaluated by the amount of sulfate groups per gram of NPs as previously described [28]. Briefly, sulfate content was obtained by formation of methylene blue after acidic hydrolysis of the samples, reduction of sulfate as hydrogen sulphide, and formation of methylene blue from *N*,*N*-dimethyl phenylene diamine dihydrochloride in a strongly acidic medium in presence of ferric chloride. The suspension of NPs (100 to 200 µL) was added to 5 mL of a reducing mixture prepared with 100 µL of concentrated hydriodic acid, 25 µL of glacial acetic acid and 2.5 g of hypophosphorous acid. The mixture was refluxed for 20 min through a water-cooled condenser under a stream of N_2_ (100 mL min^−1^) which carried away evolved hydrogen sulphide (H_2_S). After bubbling through a gas-washing column (20 mL of tris buffer 0.1 M, pH 7.2), H2S was trapped as zinc sulphide in 30 mL of a solution of zinc acetate prepared by diluting 5 mL of 0.50 M and sodium acetate 0.10 M with 25 mL of deionized water. Eight milliliter of 16 mM ferric chloride in H_2_SO_4_ 0.1 M, and 2 mL of 3.7 mM *N*,*N*-dimethyl phenylene diamine dihydrochloride in H_2_SO_4_ 9 M were added to the zinc sulphide solution, and the final volume was adjusted to 50 mL with deionized water. The vial was maintained at room temperature under dark for 20 min and the absorption was measured at 665 nm with a UV-visible spectrophotometer mc2 (Safas, Monaco-Ville, Monaco). The amount of sulphur was determined from a standard curve obtained with potassium sulfate solutions submitted to the overall process.

### 3.5. X-Ray Diffractometry

The equipment used for these measurements is an INEL EQUINOX 1000 X-ray diffractometer in asymmetric geometry (INEL, Artenay, France). The X-ray monochromatic incident beam (Co Kα1 = 0.1788976 nm radiation) makes an angle of around 6° with the sample. Two crossed slits (0.15 × 5 mm^2^) are placed through the beam at 70 mm before the sample. A continuous rotation of the sample (around the normal of the analyzed face) is done during the acquisition time in order to reduce texture effects. Line profiles are collected on a curved linear detector (0°–115°) placed at 180 mm from the sample. In order to characterize the different phases (lattice parameters, microstructures, % of phases) Rietveld refinements were performed using MAUD software v2.84 (Material Analysis Using Diffraction, University of Trento, Trento, Italy).

### 3.6. Transmission Electronic Microscopy

HR-TEM images were taken on a JEOL JEM-ARM200F Cs-corrected Field Emission Transmission Electron Microscope (Jeol, Akishima, Tokyo, Japan). Samples were prepared by dropping of the colloidal suspension onto holey carbon-coated Cu grid. The TEM images were analyzed manually using the graphical software ImageJ v1.51h (open source, http://rsb.info.nih.gov/ij/) with measurement of about 200 NPs for each sample.

### 3.7. High-Resolution Transmission Electron Microscopy and Energy-Dispersive X-Ray Spectroscopy Analysis

Samples were conventionally deposited on a carbon-coated copper grid. High-resolution transmission electron microscopy (HR-TEM) imaging and energy-dispersive X-ray spectroscopy (EDS) elemental analysis were performed on a JEOL-2100F TEM (Jeol, Akishima, Tokyo, Japan) (cold-field-emission gun, 200 kV). EDS data processing and analysis were carried out using the Thermo Scientific Pathfinder X-ray Microanalysis Software (ThermoFischer Scientific, Waltham, MA, USA).

### 3.8. FT-IR Spectroscopy

The mixture of 1 mg of NPs powder and 100 mg of potassium bromide (KBr) was ground into fine powder. The powder then is left drying at 70 °C overnight. Finally, the powder pressed by a hydraulic compressor with an approximately 10-ton force made 13 mm pellets. All of the measurements were recorded by NICOLET 380 FT-IR (ThermoFischer, Villebon-sur-Yvette, France).

### 3.9. Thermogravimetric Analysis

The weight of the coatings was estimated by thermogravimetric analysis using a LabsSys evo TG-DTA-DSC 16000 device (Setaram Instrumentation, Caluire-et-Cuire, France). About 15 mg of coated NPs was put in a furnace tube. The temperature of the furnace was controlled from 30 °C to 1500 °C.

### 3.10. Particle Size Analysis and Isoelectric Point Analysis

The hydrodynamic diameter of particles in PBS and the zeta potentials were measured by dynamic light scattering (DLS), using a Zetasizer Nano S. Size (Malvern Instruments, Orsay, UK) measurements range from 0.3 nm to 10 microns (diameter). Zeta potential dependence on pH was obtained by measuring the zeta potential in aqueous solution by adjusting the pH value by the addition of HCl and NaOH (1 M). For measurements, the folded capillary cell is filled up with 1 mL of NPs in water (C_Fe_ ~1 mM). The hydrodynamic diameter and the zeta potentials are measured at 25 °C.

### 3.11. Magnetic Properties

The magnetic behavior of the as-synthesized nanoparticles was characterized at room temperature using a vibrating sample magnetometer, VSM (Quantum Design, San Diego, CA, USA). The VSM measures the magnetization by cycling the applied field from −30 kOe to +30 kOe, with a step rate of 100 Oe/s.

### 3.12. Relaxivity

The relaxation times T_1_ and T_2_ were measured with the small animal 7T MRI Bruker Pharmascan (Bruker, Palaiseau, France). The relaxation rates of NPs in suspension are obtained by linear fitting relaxation rates (1/T_1_) and (1/T_2_) versus different Fe concentrations. The 500 µL tubes were carefully filled up with colloidal NPs suspension (CFe = 0.0261, 0.0522, 0.0774, 0.113, 0.147, 0.303, and 0.7 mM or 1.46, 2.93, 4.39, 6.32, 8.26, 17.0, and 3.92 mg Fe/L).

### 3.13. UV-Visible Spectroscopy

The absorption spectra were recorded by JASCO V-630 UV-visible spectrophotometer (JASCO, Lisses, France). Fluorescence spectra were measured by FluoroMax Plus (HORIBA Scientific, Longjumeau, France). Spectra of uncoated samples were recorded in ethanol. Spectra of coated samples were recorded in water.

### 3.14. Cell Experiments

Human umbilical vein endothelial cells (HUVECs) were obtained from ATCC (CRL 1730). Cells were cultured in Dulbecco minimum essential medium-l-glutamine (DMEM), supplemented with 10% (*v*/*v*) fetal bovine serum (FBS) and 1% penicillin-streptomycin-amphotericin (PSA). Cells were seeded in a T75 cell culture flask and kept under controlled conditions (5% CO_2_ at 37 °C) throughout the whole experiment. Cell viability was assessed using a Resazurin assay. Briefly, cells were seeded in 96-well plate (2500/well) and cultured overnight to assure cell adhesion. Then, cell culture was removed and cells were treated with four different concentrations of nanoparticles dispersed in cell culture medium (1, 0.5, 0.1 and 0.01 mg NP; *n* = 3; 100 μL/well). NP-0.50 and fuco-0.50 have been assayed. After 24 h, 48 h and 72 h cells supernatant was removed, cells were washed once with PBS and then incubated with 100 μL/well of a solution containing 10% of resazurin in the cell culture medium. 1.5 h later the optical density was recorded at 560 nm (590 nm reference wavelength; i-control microplate reader software, TECAN Männedorf, Switzerland). Cells treated with culture medium were included as negative control. A standard curve of cells was used to calculate cell number. For statistical analysis, three independent experiments were performed.

Cell morphology was studied after staining with phalloidin-TRITC and DAPI, to observe actin fibers and nuclei respectively with fuco-0.50 at 0.01 mg/mL. In brief, cells were fixed in 4% paraformaldehyde, washed three times with PBS and permeabilized using 0.1% Triton X100 for 30 min at room temperature. Then, the cells were washed with PBS and incubated in a solution containing 50 μg/mL of phalloidin-TRITC and 1 μg/mL DAPI for 30 min at room temperature. Cells were then washed and observed using a fluorescence microscope ZEISS ApoTome.2 (ZEISS, Marly-le-roi, France). Images were treated with Fiji^®^ software (ImageJ package, GPLv2, open source http://rsb.info.nih.gov/ij/).

### 3.15. Animal Study

We performed investigations on rat abdominal aorta. The procedure and the animal care complied with the ‘Principles of animal care’ formulated by the EU (Animal Facility Agreement 75-18-03; 2005), and animal experimentation was performed under the authorization 75-101 of the French Ministry of Agriculture after approval by the University ethical committee (apafis #4893-2016041112026589). Induction of the aneurysmal thrombus - Under intraperitoneal pentobarbital anesthesia (4 mg/100 g bodyweight; Ceva Santé Animale, Paris, France), approximately 20 mm of the infrarenal aorta (beginning 2 mm below the left renal artery) was separated from the vena cava. Collateral arteries were dissected from surrounding connective tissue, ligated in two places and cut between them. The aorta was clamped and four units of porcine pancreatic elastase (E-1250; Sigma-Aldrich, Lesquin, France) in 550 μL NaCl 9% was perfused transmurally during 1 h, using an automatic pressure perfusion pump. The segment was then rinsed, the catheter removed, the entry hole closed by suture and flow was re-established 26. A total of 12 male 7-week-old Wistar rats from CERJ (Le Genest-Saint Isle, France) were prepared. To localize the treated segment during the MRI session, the distances between the upper and lower points of the perfused segment and the origin of the left renal artery were measured with the microscope eyepiece, and surgical wounds were closed. This model is characterized by the presence of an intraluminal thrombus 1–3 weeks after aneurysm induction. In order to increase the size and the activity of the aneurysmal thrombus, injections of Porphyromonas gingivalis have been performed at day 8 and day 16.

### 3.16. MRI Session

Rats were anesthetized with isofluorane before injection of the NPs in the vein of the penis with a dose of 200 µmoles Fe per kg, corresponding to a plasma concentration of about 5.3 mM (average rat weight and a plasma volume of 400 g and 15 mL respectively). They were scanned with a 7 T small animal MRI (Bruker, Germany) before and after injection using a dedicated coil. For black-blood high-resolution imaging of the aortic vessel wall, a T2 sequence with cardiac gating was used with the following parameter: minimum TR = 600 ms, TE = 8 ms, FOV = 5 cm and a matrix size of 256 × 256. Continuous slices (slice thickness = 1 mm) were made under the renal aorta bifurcation. Image analysis was performed using OsiriX software (DICOM reader v3.7, Image Information Systems, Rostock, Germany). Maximum luminal narrowing was quantified by the percentage reduction (ΔR%) of the aortic luminal area. Arterial wall contrast agent uptake analysis was performed on slices that corresponded to histological sections. Intraluminal areas that evidenced visual intraluminal hypersignal from 30 min to 1 h after injection of a contrast agent were manually contoured for quantitative signal analysis. Regions of interest were pasted on all corresponding MR images. The signal-to-noise ratio (SNR) of aortic wall thrombus was measured by calculating the average signal intensity in the region of interest from MR images at each imaging point (SNR = [SIaortic wall − SImuscle]/SDnoise signal). Normalized signal enhancement (ΔNSE% = (SNRt − SNRbefore)/SNRbefore × 100) was calculated at 10, 30, 50, 75, 100 and 125 min after injection and analyses were performed between each scan time.

### 3.17. Optical Imaging

At the end of the MRI session, animals were sacrificed and the perfused portion of the aorta, including the dilatation, was harvested and flushed with PBS. Then the aorta was cut transversally into 2-mm width tissue rings which were fixed in paraformaldehyde 4% and frozen at −20 °C in cold isopentane. Healthy artery parts were used as standard samples. The aorta samples were cryo-sectioned at 10 μm thickness and placed on quartz slices for optical imaging evaluation.

## 4. Conclusions

The novel strategy developed in this work has made it possible to easily and quantitatively produce zinc oxide nanoparticles doped with iron. NPs with the iron/zinc ratio of 0.50 (NP-0.50) represented the best compromise between magnetic and optical properties and were coated with a low molecular weight fucoidan. The absence of cytotoxicity of fucoidan-coated NP-0.50 was demonstrated for concentrations up to a concentration of 0.1 mg/mL for 24–48 h, making them potential contrast agents for bimodal imaging using MRI and fluorescence microscopy. The mechanism involved in the formation of the NPs remains to be fully clarified since a mixture of cubic and wurtzite phases was obtained for NP-0.50. However, we consider these first results as promising for further development of such nanomaterial for preclinical studies with dedicated animal models This optimization would implicate not only to master the surface chemistry of the NPs but also the development of optical imaging setup allowing 3D images reconstruction.

## Figures and Tables

**Table 1:** Physicochemical features of Zn(Fe)O bare nanoparticles (NPs). Composition, size, cell parameters and proposed stoichiometry. Rietveld refinements for NPs with R_Fe_ = 0.35 and 0.50 have been performed with the stoichiometry established from chemical analysis. For R_Fe,i_ = 0.50, weight ratio of crystal phases = W/S = 55.1/44.9, volume ratio of crystal phases = 33.4/66.6.

R_Fe,i_	R_Fe,f_^a^	TEM		XRD		Stoichiometry ^b^
		Size (nm)	PDI	Size (nm)	Lattice Parameters	
0.05	0.06	7.0 ± 0.8	0.11	5.9 ± 0.2	Wurtzite a: 0.325839 ± 6.68 × 10^−5^ c: 0.53225 ± 2.05 × 10^−5^	Fe_0.06_Zn_0.94_O
0.15	0.18	7.0 ± 1.0	0.14	6.7 ± 0.2	Wurtzite a: 0.327126 ± 4.6 × 10^−5^ nm c: 0.52281 ± 1.156 × 10^−5^ nm	Fe_0.18_Zn_0.82_O
0.35	0.42	6.3 ± 0.2	0.03	5.8 ± 0.2	Wurtzite a: 0.327094 ± 2.83 × 10^−5^ nm c: 0.531681 ± 2.83 × 10^−5^ nm	Fe_0.42_Zn_0.58_O
0.50	0.57	4.2 ± 0.6	0.14	4.1 ± 0.1	Wurtzite a: 0.32499 ± 1.78 × 10^−4^ nm c: 0.530024 ± 3.87 × 10^−4^ nm	Fe_0.54_Zn_0.46_O ^c^ 44.9% (*w*/*w*)
				3.8 ± 0.4	Cubic a: 0.84752 ± 1.15 × 10^−4^ nm	ZnFe_2_O_4_^d^ 55.1% (*w*/*w*)

^a^ obtained by atomic absorption (±0.01), ^b^ see ESI for the determination of stoichiometry, ^c^ wurtzite phase (W), ^d^ spinel phase (S).

## Supplementary Materials

The following are available online. Figure S1. Experimental setup for synthesis of NPs with Dean-Stark apparatus. Figure S2. EDS analysis of NP-0.35 and NP-0.50. Figure S3. UV-visible absorption spectra of the coating polymers: carboxymethyl dextran (CMD) and fucoidan in water. Figure S4. MTT proliferation assay of NP-0.50 and fuco-0.50 (mg/mL) toward human vascular endothelial cells (HUVECs) after 24 h and 72 h. Figure S5. Fluorescence microscopy setup. Figure S6. Transversal image of rat abdomen centered on the abdominal aorta and time course of contrast enhancement over 2 h after injection of fuco-0.50. The red dot circle depicts the area where contrast was increasing, corresponding to the vascular wall where thrombosis occurred. Figure S7. MR signal vs time after CMD-coated and fucoidan-coated NPs injection on T_2_* weighted MR images with the same animal.
