# Supplementary material for: Bimodal Fucoidan-Coated Zinc Oxide/Iron Oxide-Based Nanoparticles for the Imaging of Atherothrombosis
Source: Molecules. 2019 Mar 8;24(5):962. doi: 10.3390/molecules24050962 (PMC6429451; doi:10.3390/molecules24050962)
Supplement: Supplementary file 1 [file molecules-24-00962-s001.pdf]

Electronic Supplementary Material (ESI) for Molecules

Bimodal Fucoidan-Coated Zinc Oxide/Iron Oxide-Based Nanoparticles for the Imaging of Atherothrombosis

Hoang Nguyen [1,2], Eric Tinet [2], Thierry Chauveau [3], Frédéric Geinguenaud [1], Yoann Lalatonne [1,4], Aude Michel [5], Rachida Aid-Launais [1,6], Clément Journé [1,6], Caroline Lefèbvre [7], Teresa Simon-Yarza [1], Laurence Motte [1], Noureddine Jouini [3], Jean-Michel Tualle [2] and Frédéric Chaubet [1,*]

[1] Laboratory for Vascular Translational Science, Inserm U1148, Institut Galilée – Université Paris Diderot, Université Paris 13, Sorbonne-Paris-Cité, 99 av JB Clément, 93430 Villetaneuse, France; ndhoang@iop.vast.ac.vn (H.N.); frederic.geinguenaud@univ-paris13.fr (F.G.); yoann.lalatonne@aphp.fr (Y.L.); rachida.aid@inserm.fr (R.A.-L.); clement.journe@inserm.fr (C.J.); teresasimonyarza@gmail.com (T.S.-Y.); laurence.motte@univ-paris13.fr (L.M.)

[2] Laboratoire de Physique des Lasers, UMR CNRS 7538, Institut Galilée - Université Paris 13, Sorbonne-Paris-Cité, 99 av JB Clément, 93430 Villetaneuse, France; eric.tinet@univ-paris13.fr (E.T.); tualle@univ-paris13.fr (J.-M.T.)

[3] Laboratoire des Sciences des Procédés et des Matériaux, UPR CNRS 3407, Institut Galilée - Université Paris 13, Sorbonne-Paris-Cité, 99 av JB Clément, 93430 Villetaneuse, France; thierry.chauveau@univ-paris13.fr (T.C.); jouini@univ-paris13.fr (N.J.)

[4] Service de Médecine Nucléaire, Hôpital Avicenne Assistance Publique-Hôpitaux de Paris, F-93009 Bobigny, France

[5] Laboratoire Phénix, UMR 8234, UPMC, 4 place Jussieu, 75252 Paris Cedex 05, France; aude.michel@upmc.fr

[6] Fédération de Recherche en Imagerie multimodalité (FRIM), UMS 34, Hôpital Bichat, 46 rue Henri Huchard, 75018 Paris Cedex, France

[7] Université de Technologie de Compiègne, Service d'Analyse Physico-Chimique, Direction à la Recherche, Rue du Dr Schweitzer, CS 60319, 60203 Compiègne cedex, France; caroline.lefebvre@utc.fr

* Correspondence: frederic.chaubet@univ-paris13.fr; Tel: +33-1-49-40-40-90; Fax: +33-1-49-40-30-83

Received: 2 February 2019; Accepted: 6 March 2019; Published: 8 March 2019

Exprimental setup

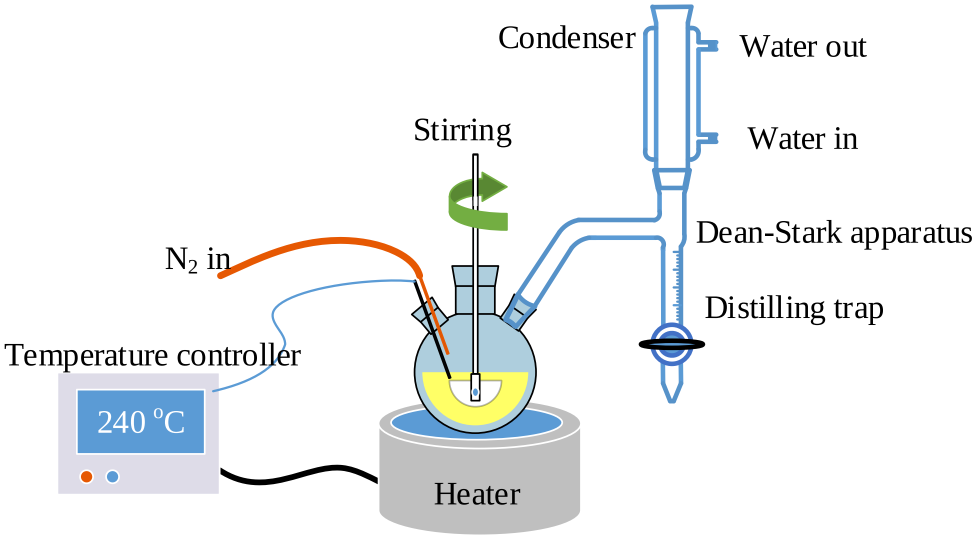

Figure S1. Experimental setup for synthesis of NPs with Dean-Stark apparatus.

Stoichiometry

The formation of Zn(Fe)O crystals without modification of the elementary cell, *ie* leading to the same crystal lattice, is obtained either by substitution of some zinc ions by iron ions, or by inclusion of iron ions into tetraedric sites of the hexagonal cell (see manuscript). For $R_{Fe,i}$ = 0.50, we tried to propose a stoechiometry for the hexagonal phase by making the hypothesis of substitution. In this case there is a simple relation between iron and zinc content: $Zn_x(Fe)_yO$ with x+y=1

Let us assume that the number of mol $Zn_x(Fe)_yO$ and $ZnFe_2O_4$ is labelled by a and b, respectively.

From XRD data, we found that the weight ratio $Zn_x(Fe)_yO$ / $ZnFe_2O_4$ is 0.449:0.541 (table 2.2). From AAS data, the ratio between the number of mol of Zn and of Fe in $R_{Fe,\,i}$ = 0.50 would be 0.45:0.55.

Hence, we can evaluate x and y by solving the system of equations 2.1:

$$\begin{cases} x + y = 1 \\ \frac{(65x+56y+16)a}{(65+168+48)b} = \frac{0.45}{0.55} \\ \frac{xa+b}{ya+2b} = \frac{0.43}{0.57} \end{cases} \quad (2.1)$$

In this system, the first equation accounts for the fact that Fe doping in ZnO takes place by substitutions of Zn by Fe, the second equation accounts for the 0.45:0.55 weight ratio between both phases, and the third equation accounts for the ratio of the number of mol of Zn and of Fe. Finally, we have:

$$\begin{cases} a = 2.55b \\ x = 0.54 \\ y = 0.46 \end{cases} \quad (2.2)$$

From the calculated results, we know that NP-0.50 is the mixture of $Zn_{0.54}(Fe)_{0.46}O$ and $ZnFe_2O_4$, and the ratio between quantities of $Zn_{0.54}(Fe)_{0.46}O$ and $ZnFe_2O_4$ is 2.55:1. The ratio of Zn and Fe in $Zn_x(Fe)_yO$ phase is rather similar to the initial composition. In addition, the beginning of formation of $ZnFe_2O_4$ in this sample can be observed.

EDS

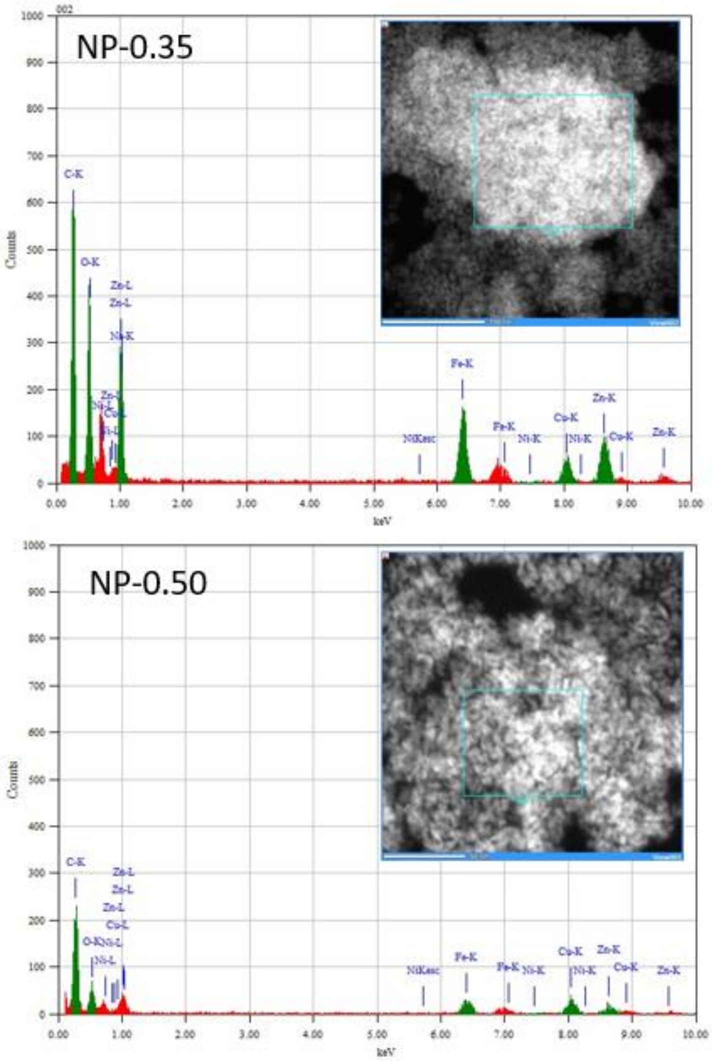

Figure S2. EDS analysis of NP-0.35 and NP-0.50

Absorption spectra of capping polysaccharides

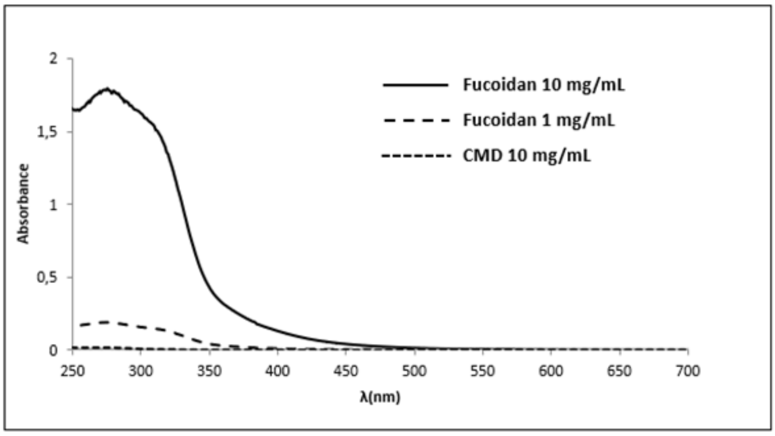

Figure S3. UV-visible absorption spectra of the coating polymers: carboxymethyl dextran (CMD) and fucoidan in water.

Cytotoxicity

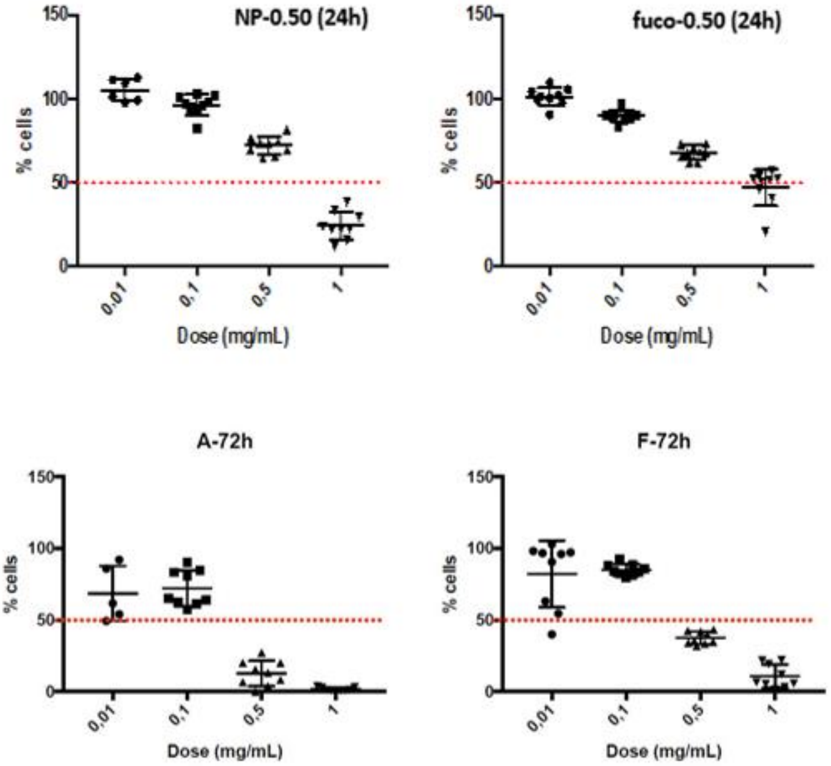

Figure S4. MTT proliferation assay of NP-0.50 and fuco-0.50 (mg/mL) toward human vascular endothelial cells (HUVECs) after 24h and 72h

Optical imaging

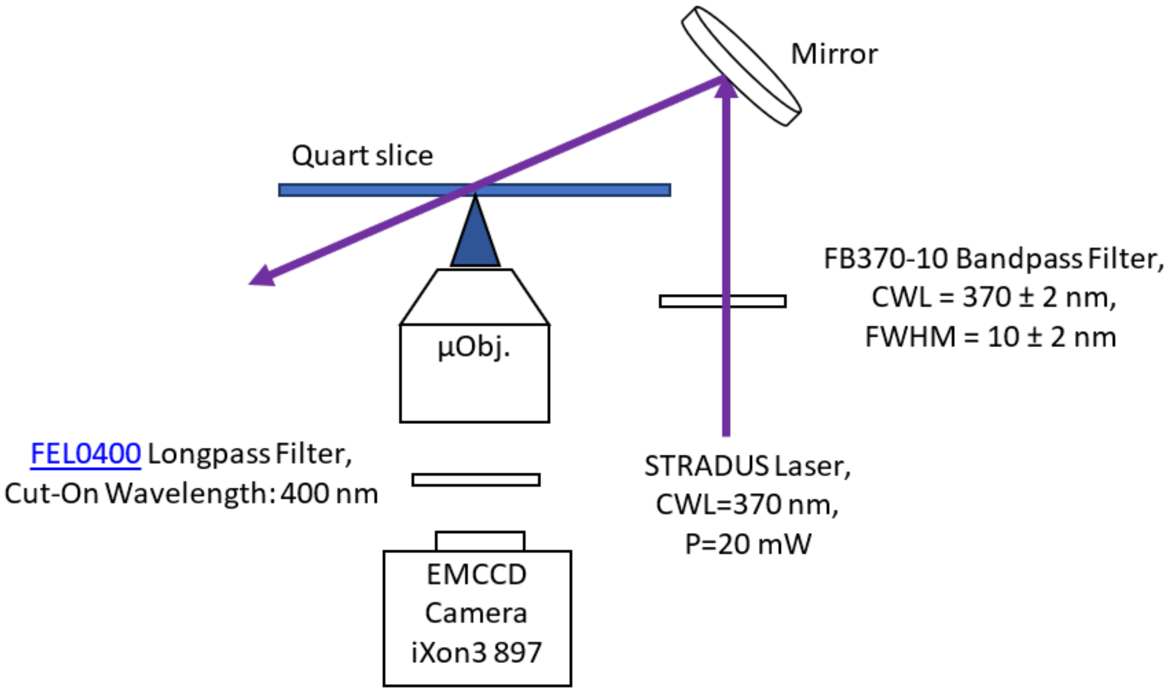

Figure S5. Fluorescence microscopy setup.

A 20 mW UV (370 nm) diode laser (Laser Stradus, Vortran Medical Technology) is the light source. A filter cuts off the unwanted photons from the laser source: the laser indeed has some internal parasitic fluorescence, which could be seen on the fluorescence spectrum. An aluminium mirror directs the laser beam towards the sample. Another filter stops the laser beam from hitting the CCD camera. A micro objective collects fluorescence signal from the sample.

MRI

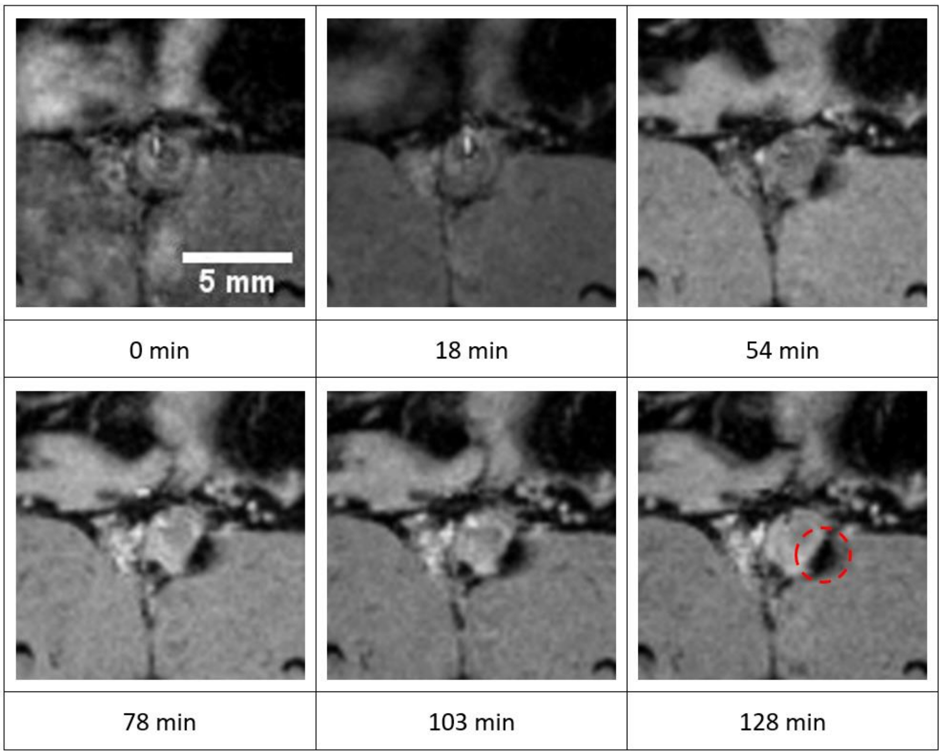

Figure S6. Transversal image of rat abdomen centered on the abdominal aorta and time course of contrast enhancement over 2 hours after injection of fuco-0.50. The red dot circle depicts the area where contrast was increasing, corresponding to the vascular wall where thrombosis occurred.

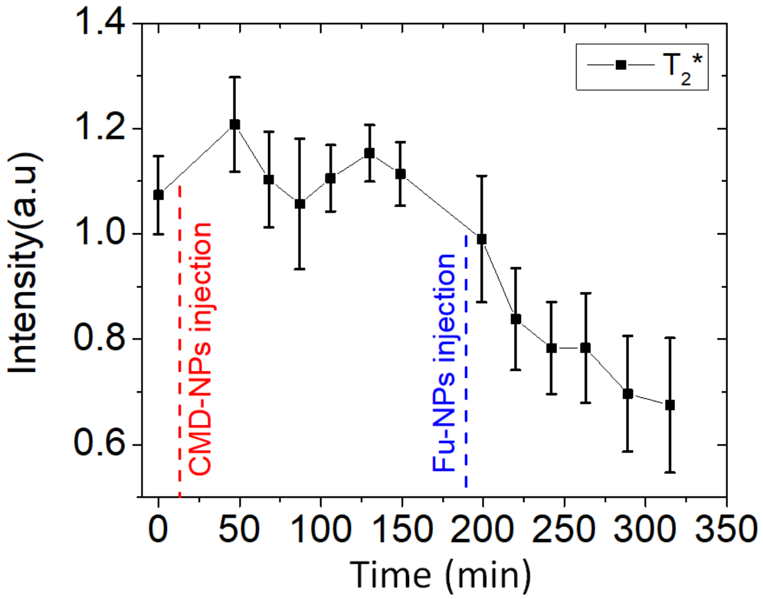

Figure S7. MR signal vs time after CMD-coated and fucoidan-coated NPs injection on T_2* weighted MR images with the same animal.
